# How to copy and paste DNA microarrays

**DOI:** 10.1038/s41598-019-50371-1

**Published:** 2019-09-26

**Authors:** Stefan D. Krämer, Johannes Wöhrle, Philipp A. Meyer, Gerald A. Urban, Günter Roth

**Affiliations:** 1 ZBSA – Center for Biological Systems Analysis, Albert-Ludwigs-University Freiburg, Habsburgerstrasse. 49, D-79104 Freiburg, Germany; 2 Faculty for Biology, Albert-Ludwigs-University Freiburg, Schaenzlestrasse 1, D-79104 Freiburg, Germany; 3 IMTEK – Dep. of Microsystems Engineering, Albert-Ludwigs-University Freiburg, Georges-Köhler-Allee 103, D-79110 Freiburg, Germany; 4 BioCopy GmbH, Spechtweg 25, D-79110 Freiburg, Germany; 5 BIOSS – Center for Biological Signalling Studies, Albert-Ludwigs-University Freiburg, Schaenzlestrasse 18, D-79104 Freiburg, Germany; 6 BioCopy Holding AG, Industriestrasse 15, 8355 Aadorf, Switzerland

**Keywords:** DNA, DNA probes, Kinetics

## Abstract

Analogous to a photocopier, we developed a DNA microarray copy technique and were able to copy patterned original DNA microarrays. With this process the appearance of the copied DNA microarray can also be altered compared to the original by producing copies of different resolutions. As a homage to the very first photocopy made by Chester Charlson and Otto Kornei, we performed a lookalike DNA microarray copy exactly 80 years later. Those copies were also used for label-free real-time kinetic binding assays of apo-dCas9 to double stranded DNA and of thrombin to single stranded DNA. Since each DNA microarray copy was made with only 5 µl of spPCR mix, the whole process is cost-efficient. Hence, our DNA microarray copier has a great potential for becoming a standard lab tool.

## Introduction

DNA microarrays are small structures on surfaces which are comprised of hundreds to thousands of individual DNA spots. They can be used for a variety of applications, such as gene expression profiling, transcription factor binding assays and genotyping^1,2^. Yet, the generation of microarrays is still cumbersome. Up to now, the two main methods to produce them are spotting^3^ and *in-situ* light directed synthesis (photolithography)^4^. Either way, the arrays are manufactured from scratch. For DNA microarray spotting, the individual DNA sequences need to be chemically synthesized or produced via cell culture in advance. However, when choosing chemical synthesis, the DNA length is limited to short oligomers because of increasing synthesis errors. Hence, for longer DNA sequences, cell cultures and a high effort of DNA cloning are often unavoidable. This fact makes the creation of microarrays with many spots expensive. Using photolithography, the DNA microarrays are directly synthesized onto the chip. Consequently, only the four different DNA nucleotides are needed. However, lithographic DNA microarrays are even more strongly restricted by the same sequence length limitations than chemical DNA synthesis. Hence, it is only useful for applications working with short DNA sequences^2^.

Summing up, building DNA microarrays from scratch every time is expensive, time consuming and often needs high-tech equipment to achieve the necessary reliability. However, if a DNA template is already available, the most effective way to replicate these DNA molecules is through the natural DNA replication reaction catalysed by DNA polymerases. The speed and precision of DNA polymerases are still unrivalled by chemical synthesis and will remain very much so for a long time^5^. Previous works have already demonstrated that polymerases can be utilized to replicate DNA microarrays^6–13^. However, none of them were able to perform a copy from a glass substrate to another glass substrate. Yet, since DNA microarrays are often spotted or synthesized onto glass, this can be an obstacle. Furthermore, these replication techniques are based on the de-hybridization of complementary DNA molecules. This only allows the generation of a complementary DNA microarray, representing a negative of the original DNA array.

In this work, we describe an alternative technique to copy original DNA microarrays, which is comparable to that of a standard office copier. The original DNA microarrays used were produced by classic spotting and are comprised of many little spots. During the copy process, those spots become pixelated and the smallest feature which is transferred to the copy is defined as a dot. Hence, the original DNA microarrays are comprised of spots, whereas the copied DNA microarrays consist of dots. Depending on the applied copy resolution, one spot of an original microarray can result in multiple dots in the corresponding microarray copy.

## Results

### Microarray copying of printed DNA microarrays

**Figure 1:**
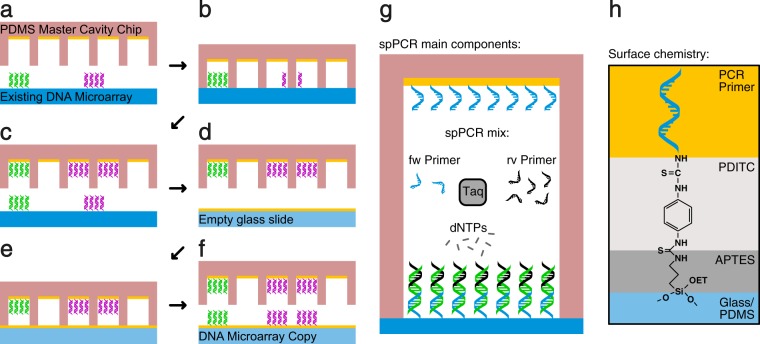
The principle of DNA microarray copying. (**a**) A PDMS master cavity chip coated with primer is filled with spPCR mix and placed on top of an original DNA microarray consisting of two different DNA species (magenta and green). (**b**) After closing, a first spPCR is performed to amplify the DNA and (**c**) to attach it to the inside of the cavities. (**d**) The cavity chip is washed, blocked, and refilled with fresh spPCR mix. (**e**) The cavity chip is placed on top of an empty glass slide coated with primer and a second spPCR is performed. (**f**) After the spPCR, the cavity chip is opened, revealing a copy of the original DNA microarray. The position, size and number of the cavities limits the spatial resolution of the copy. In this example, the green DNA spot is enclosed by one cavity resulting in one dot in the copy. The magenta DNA spot is enclosed by two cavities, hence creating two magenta DNA dots. (**g**) Illustration of the spPCR components. One PCR primer species is attached to the surface. After an initial amplification in liquid phase, the PCR reaction is forced to the surface. (**h**) Illustration of the chemical composition of the surface. The individual layers are indicated using different colors.

We used solid phase polymerase chain reaction (spPCR) to replicate the DNA and a cavity chip to maintain the spatial order in our DNA microarray copying process. In a first spPCR the DNA from the original DNA array is copied into cavities (Fig. 1a–c) and in a second spPCR the DNA is copied back from the cavities onto a new surface (Fig. 1d–f). The resulting DNA microarray is now a copy of the original microarray consisting of the same DNA and comprising the same spatial information. The spPCR reaction used in our experiments is similar to Hoffman *et al*. 2012 (Fig. 1g). Moreover, the surface primer used to amplify the DNA molecules on the surface were immobilised with help of the well described PDITC chemistry (Fig. 1h)^14,15^.

**Figure 2:**
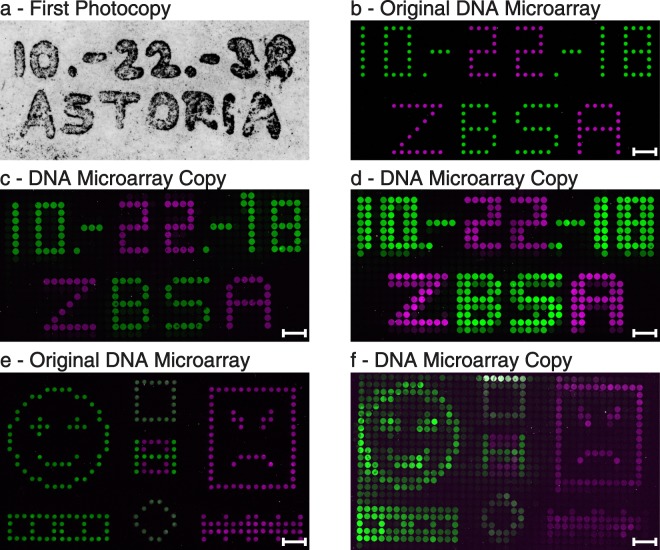
Examples of DNA microarray copies. (**a**) The very first photocopy made by Chester Charlson (Courtesy of Xerox Corporation). (**b**) Our lookalike spotted original DNA microarray containing date and institute name. (**c**) A DNA microarray copy of the original DNA microarray with a perfect alignment of DNA spots and cavities. (**d**) Another DNA microarray copy, but with a misalignment of DNA spots and cavities, generating a blurred copy. (**e**) A spotted original DNA copy comprised of a happy and an angry smiley with additional structures. (**f**) The corresponding copy of the original smiley array. (**c,d,f**) Image contrasts were enhanced by a factor of 4 in the green channel and by a factor of 7 in the magenta channel for better visualization. The scale bars show a length of 1 mm. All illustrated copies were produced using cavity chip 2 (Supplementary Table S1), with cavity diameters of 300 µm and a cavity distance of 50 µm. Spotted original spots have a diameter of 180 µm and a spot distance of 170 µm.

To demonstrate the working principle of our DNA copier, we produced original DNA microarrays by printing a simple pattern consisting of two different single stranded DNA molecules (magenta: 89 bp; green: 103 bp). We have chosen our spotting scheme to resemble the style of the first photocopy by Chester Charlson and Otto Kornei and performed the copy exactly 80 years later. Similar to Charlson’s first copy (Fig. 2a), we spotted the date of our copy experiment and the name of our institute (Fig. 2b). We used PDMS master cavity chips that had approximately the same cavity dimensions as the spots of the original DNA arrays. Yet the overall chip area dimensions are larger (Supplementary Table S1). Hence, the chip was manually placed onto the area of the microarray without further precautions about alignment. This scanning process results in a random alignment of individual cavities to individual, original spots. Even without a perfect alignment, the image will be recreated depending on the resolution (in this case the cavity number and diameters). After the copy experiment, all DNA microarrays – the originals as well as the copies – were hybridised using fluorescently labelled DNA probes, resulting in a two-colour image for each array. In both illustrated copies (Fig. 2c,d) the date as well as the institute name are clearly readable. In addition, the arrangement of the green and magenta DNA dots matches the spots of the original array (Fig. 2b). The measurement of the signal intensities of the copied dots revealed lower values by a factor of 3.98 ± 0.41 (n = 3) in the green and by a factor of 8.64 ± 2.55 (n = 3) in the magenta channel (Supplementary Fig. S3). If original DNA spots and the cavities of the master cavity chip are in perfect alignment, a nearly identical spatial image is created (Fig. 2c). If the cavities of the chip are misaligned, a blurred but still readable copy is generated (Fig. 2d). For a more complex DNA image, we used the same DNA sequences to print smileys (Fig. 2e) and subsequently generated a copy (Fig. 2f).

### Resolution and moiré patterns

**Figure 3:**
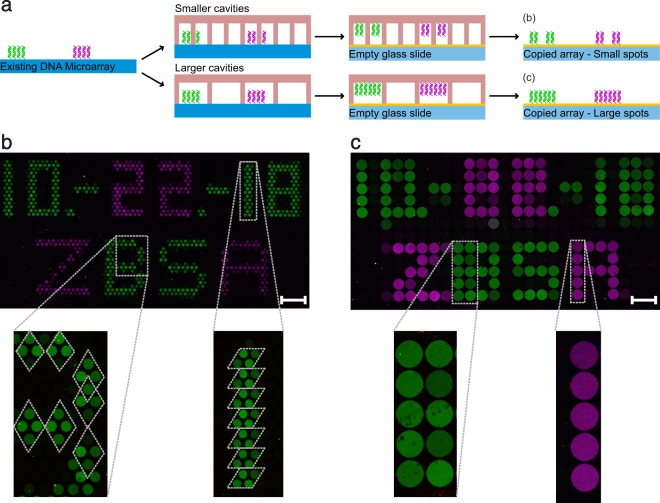
DNA microarray copies can also be performed with altered resolutions. (**a**) Schematic representation of the performed microarray copy approach. The principles are similar to the method shown in Fig. 1a–f. However, this time cavity chips were used with smaller (**b**) or larger (**c**) cavity diameters compared to the original spot sizes. (**b**) Hybridised fluorescent image of the small cavity (150 µm) copy with enlarged sections. (**c)** Hybridised fluorescent image of the large cavity (500 µm) copy with enlarged sections. Dotted lines in the enlarged pictures illustrate the Moiré pattern. The scale bars show a length of 1 mm. Cavity chip type 1 was used for the high resolution copy (**b**) and cavity chip type 3 was used for the lower resolution copy (**c**).

Moiré patterns are interference patterns which can be observed when a grid is placed on top of another grid^16^. Especially if the grids differ in spatial resolution or grid pattern (e.g. quadratic vs. hexagonal), new shapes can be created. The appearance of Moiré patterns can be used to generate DNA microarray copies with altered dot patters compared to the one of the original DNA microarrays. For this purpose, we performed DNA microarray copies using cavity chips of higher (1.3 times smaller cavity diameter) as well as lower spatial resolution (2.8 times larger cavity diameter) than the spots of the original DNA microarray (Supplementary Table S1). Again, we used the same microarray pattern as before (Fig. 2b). In case of the high-resolution copy (Fig. 3b), the cavities of the PDMS master cavity chip are arranged in a hexagonal, honeycomb-like pattern. Depending on the alignment of the cavity chip on the original DNA microarray, each DNA spot generates a Moiré pattern of either vertical or flat rhombuses (Fig. 3b, enlarged sections). Here, about four dots of the copy are generated by one spot of the original DNA microarray. Choosing a lower resolution master cavity chip resulted in a smeared and blurred copy (Fig. 3c). Here, about seven spots of the original array are depicted by five dots of the copy. The chosen spatial resolution of the cavity ensured that the original spots could not get in contact with more than two cavities. Therefore, depending on how the master cavity chip was aligned on the original DNA microarray, either one or two larger DNA spots can be observed in the corresponding copy (Fig. 3c, enlarged sections).

### Dot merging

**Figure 4:**
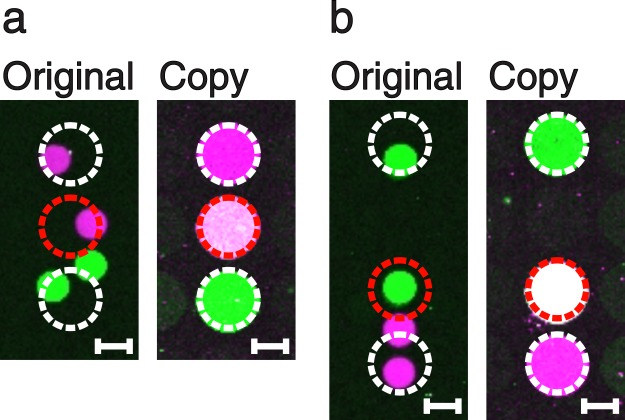
Two examples of spot merging (**a**,**b**). The original DNA microarrays (left) were produced by digital solid phase PCR using our PDMS master cavity chips. Thereafter, copies of lower resolution were performed (right). In each case, the middle dots (red dashed circle) of the copies show a pink to white colour, indicating that they are mixtures of both, the magenta and green DNA species. Scale bars show a length of 100 µm.

Depending on the original DNA microarray spot sizes, distances and the spatial resolution of the master cavity chip, it is also possible to merge spots on the DNA copy into one single DNA dot. This transformation can take place when one cavity of the master cavity chip is placed on top of two spots of the original array. Hence, the spatial resolution of the master cavity chip has to be identical to or lower than the resolution of the original array. This kind of aberration can be exploited and even enforced in order to create DNA mixture spots, which were not present on the original array. We generated an original DNA microarray via a digital PCR process with a hexagonal pattern of round spots (diameter of 150 µm; distance of 50 µm), similar to a method published before^15^. Subsequently, we made a copy using a quadratic pattern of round dots (diameter of 300 µm; distance of 50 µm) featuring an about 2 times lower resolution (Fig. 4a,b). The copied dots in the middle (Fig. 4a,b, right sides) contain a mixture of the magenta and the green coloured DNA species.

### Label-free binding experiments using copied DNA microarrays

**Figure 5:**
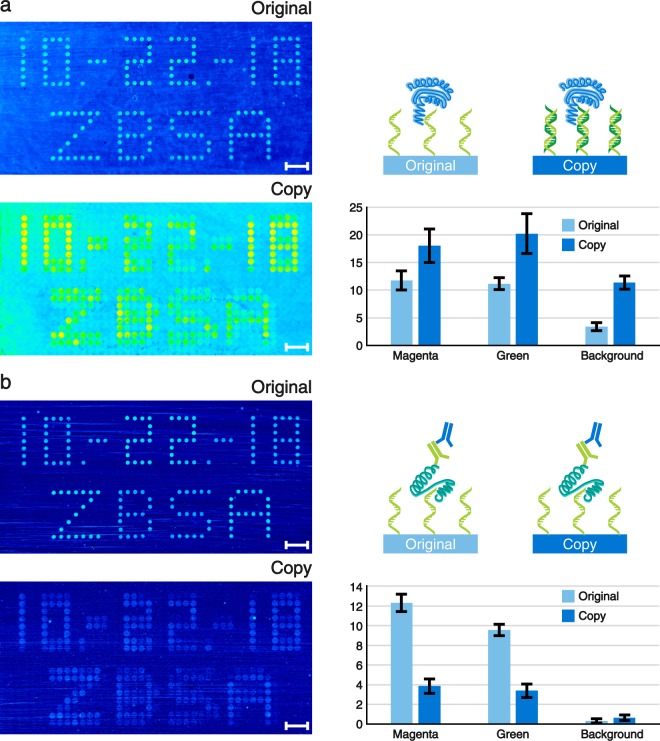
Label-free SCORE measurement pictures of (**a**) apo-dCas9 and (**b**) thrombin binding to the original and copied DNA microarrays are shown on the left. The more yellow to red the spots and dots appear, the stronger is the binding signal. Corresponding schematic representations and bar plots illustrating the average binding signals of the spots are shown to the right of each assay. The units of the bar plots are given in milliSCORE. The apo-dCas9 assay was performed using the protein only, whereas for the thrombin measurement a three step assay with thrombin followed by a primary and a secondary antibody was used. The binding signals of the spots were averaged according to their fluorescent colours (see Fig. 2). Background signals were assessed by measuring four large background areas around the array region. The values of the bar plots represent the means and the error bars show their standard deviations. N-values for (**a**): original-magenta = 244; original-green = 98; copy-magenta = 122; copy-green = 159. N-values for (**b**): original-magenta = 71; original-green = 98; copy-magenta = 59; copy-green = 183. N-values of all backgrounds equal 4. Scale bars show the length of 1 mm.

Our spotted original microarrays are made of single stranded DNA (ssDNA) molecules. By default, the copied DNA microarrays contain double stranded DNA (dsDNA) as this is the natural end product of the spPCR. Yet, using our surface chemistry, only one of the two DNA strands is covalently bonded to the surface. Hence, the copies can be de-hybridised in order to turn the dsDNA into ssDNA. Consequently, our copy approach allows us to choose between dsDNA and ssDNA microarrays. This is a feature which is not yet easily accessible with any other microarray manufacturing process. However, having both types of DNA molecules available can be particularly useful for screening purposes. As an example, we used our copied microarrays and performed an apo-dCas9 (Cas9 not able to cut DNA without crRNA) assay with the dsDNA microarray (Fig. 5a) and a thrombin assay with the ssDNA microarray (Fig. 5b). The sequences used for our copy experiments (magenta and green spots) are derived from known and well-characterized thrombin aptamers^17^. Hence, in their single stranded form they are able to bind to thrombin with a high specificity. We performed label-free single colour reflectometry (SCORE) end point measurements to evaluate the binding of the proteins^18^. Additionally, we quantified the average binding intensities of all magenta and green spots on the microarray (in accordance with Fig. 2b). Furthermore, four large areas outside of the array region were analysed for obtaining a background signal. The copied DNA microarray showed a stronger apo-dCas9 binding than the corresponding original DNA microarray, including its background (Fig. 5a). Moreover, all numbers and letters are clearly readable. No major differences in binding intensities for the magenta and green spots could be observed within the original or copied array signals.

After the apo-dCas9 assay, both DNA microarrays were de-hybridised to strip off all apo-dCas9 proteins and to generate a ssDNA array out of the dsDNA microarray copy. Our thrombin SCORE experiment was performed in three steps. First, thrombin was flushed over the DNA microarray, followed by a primary antibody, followed by a secondary antibody (Fig. 5b, schematic representation). The experiment revealed that there is indeed a good binding of thrombin and the corresponding antibodies to all spots of the original DNA microarray (Fig. 5b). The formerly magenta coloured spots were quantified to show a slightly stronger binding signal compared to the green spots. Our copied DNA microarray also showed a binding signal to thrombin and the antibodies. However, the signal is weaker compared to the original microarray but still distinguishable from the background.

Since SCORE is a real-time imaging technique to perform label-free binding experiments, we also provide videos of the measured binding events (Supplementary Movies S4 and S5).

## Discussion

The first photocopy was made on the 22^nd^ of Oct 1938, offering new possibilities to replicate texts and images. Exactly 80 years later, we performed a DNA copy, enabling us to copy a DNA image from an original DNA microarray. All microarrays were clearly readable and showed an exact effigy of its original image (Supplementary Fig. S2). Although the copies showed a lower fluorescent signal than the original arrays, the DNA sequences remained identical. Since the copy of one DNA microarray only needs 5 µl of spPCR mix, the process is cost-efficient. In addition, all DNA microarray copies initially contain dsDNA, which can be converted into ssDNA microarrays after a de-hybridisation step. Since we make use of spPCR, a perfect unidirectional orientation of the DNA molecules with a 5′ (surface) to 3′ direction can be ensured. Other photolithographic methods are also available with which such an orientation can be achieved, although a 5′-3′ orientation is not standard^19,20^. The DNA density on the spot is mainly limited by the number of primers attached to the surface, the size of the polymerase and the number of spPCR cycles used. This allows many optimization and alteration possibilities, for example an on-demand fine-tuning of the copied DNA spot densities, which is comparable to a brighter or darker photocopy.

Similar to a photocopy, we occasionally observed positive dots on the copied DNA microarrays where no template spot is encountered on the original. These errors may arise from cross contaminations which can occur prior to or during the spPCR. The first case can be caused by loose DNA strands that are distributed on the original DNA microarray and may originate from insufficient blocking or washing procedures. The second case can occur through a leakage between neighbouring cavities. Furthermore, it can happen that no DNA dot is observed where there should be one according to the original (no example shown). Those effects mainly take place because of tiny air bubbles trapped inside the cavities of the PDMS master cavity chip.

We also demonstrated that our DNA microarray copies underlie the same effects like a pixelated photocopy, including spot merging and moiré patterns. Yet, these effects may not only happen accidentally, but can also be enforced through a careful selection of the cavity sizes and layouts. They can thus be used for DNA synthesis, modifications or alterations of existing DNA microarrays on purpose. We were able to show that two different spots of the original microarray can be combined on the microarray copy. Hence, our method does not only allow the replication of existing DNA microarrays but also their alteration. We conclude, that new microarrays could be created by choosing the right PDMS master cavity chip dimensions. Depending on the right choice of primer combinations, it should theoretically be possible to combine and mix two existing source microarrays. In principle, two DNA microarrays would be copied on top of each other. This technique would allow to generate barcoded DNA microarrays, which can then be used for all kinds of transcriptomic studies. In theory, many known biochemical reactions for DNA can be applied inside the cavities of the master cavity chip to alter or modify the DNA. Having those possibilities of DNA modifications (PCR, cloning, gene editing and many others) in mind, our copies may be the “paper” which many new high-throughput applications may be “written” on.

Our SCORE experiments demonstrated that the molecules of the copied DNA microarrays are accessible for label-free binding measurements. Although the copied DNA microarrays are comprised of less DNA molecules per dot than the original array, the apo-dCas9 binding signal was much stronger for the copied array. Since the copied array contains double stranded DNA molecules after the copy process, we conclude that the apo-dCas9 protein has a higher specificity to dsDNA molecules than to ssDNA molecules. Interestingly, the background of the copied DNA microarrays is in the range of the magenta/green spots of the original array. This is because the whole microarray slide of the copy is homogenously covered with single stranded forward primer needed for the spPCR. That is not the case for the spotted microarray, which only consist of the DNA spots. Therefore, the background of the copied DNA microarray basically displays the signal of apo-dCas9 binding to single stranded DNA molecules. The performed Thrombin assay on the ssDNA microarray copy showed a specific binding to the single stranded DNA molecule aptamers. However, the Thrombin signal of the copied array is lower compared to the original array for both DNA species, which is in consistence to the hybridization results. The differences between original and copy are still subject of research. Nonetheless, these label-free screening results prove that our DNA microarray copy technique can be used for multiple screening applications.

Our DNA microarray copy method combined with the label free SCORE measurement holds a great potential for old and new applications. We think that the presented DNA copies only represent the first step of microarray copying. Since the DNA molecules of the original DNA microarray are immobilised inside the cavities of the master cavity chip, RNA^21,22^ or protein^23–26^ copies could also be realised. This can be done by changing the copy surface and biochemical mix. Such copies will open a new world of screening and synthetic biochemistry.

## Material and Methods

### Structuring of Si-wafers as master mould

For realising the desired structures in PDMS, clean standard 4″ Si-wafers were used. A wafer was heated to 130 °C for 60 s. The hot wafer was laminated as quickly as possible (ORDYL SY330 Elga Europe, Italy) according to manufacturer protocol using a Polatek laminator. Before exposure, the photolithography mask (Zitzmann GmbH, Germany with a resolution of 32,000 dpi) had to be placed in the maskaligner (MA6/BA6 Karl Suss). Masks were drawn using Solidworks (SW2013) and Coreldraw (CorelDraw X6 V16). The wafer was illuminated for 12.5 s in soft contact mode with an alignment-gap of 100 µm. Thereafter, the wafer was baked at 85 °C for 60 s. Next, the wafer was developed in ordyl-developer (Elga Europe, Italy) for about 20 s, followed by another 15 s wash in ordyl-developer. The developer times depend on the mask structures and need to be adjusted. After developing, the wafer was baked at 100 °C for 60 min. Finally, the wafer was placed in a desiccator together with a microscope slide carrying a drop of 25 μl of Trichloro(1H,1H,2H,2H-perfluorooctyl)silane (Sigma-Aldrich, Germany) for 8 hours, followed by a post bake step of 60 min at 90 °C.

### Moulding tools production

To prevent wafer damage, we developed in house moulding tools made out of aluminium. The wafers were glued to the lid using Soudal FIX ALL CRYSTAL glue (Soudal N. V., Germany). After alignment, an overnight drying step was applied. The bottom part of the tool has placeholders for the aluminium carriers. We used backbone aluminium carriers with a thickness of 1 mm, a length of 75 mm and a width of 25 mm (same dimensions as a microscope slide). We also designed them to have holes for the later fluidic connections.

### PDMS cavity chip production

Two aluminium metal sheets (standard microscope glass slide measurements), containing two 1 mm holes, were plasma activated using a plasma generator (ZEPTO, Diener electronics) for 2 min at 100 W and at a gas flow of 20 l/h. PDMS (Elastosil RT 607; Wacker Chemie, Germany) was mixed in a 9:1 ratio (component A: component B) with a speedmixer (speedmixer Series DAC 150 for 60 s at 1500 rpm). Subsequently, the metal sheets were put into our moulding forms and coated with 9 g of still liquid PDMS. The moulding form was put under vacuum in a desiccator for 6 min. Thereafter, the moulding form was closed with the lid which contains the structured wafer. Next, the closed moulding form was incubated at 60 °C for 1 h. Thereafter, the PDMS cavity chips were demoulded and microfluidic holes were punched into the PDMS using a 0.5 mm biopsy punch.

### Production of primer coupled glass slides

Glass slides (standard microscope slides as well as SCORE slides) were rinsed with acetone, isopropanol and diH_2_O. Subsequently, they were dried in an N_2_ gas stream. Prior to silanisation, slides were plasma activated using a plasma generator (ZEPTO, Diener electronics) for 1 min at 100 W and at a gas flow of 20 l/h. Thereafter, slides were put into a (3-aminopropyl)triethoxysilane (APTES, Sigma-Aldrich, Germany) solution (89% acetone [v/v], 10% dH2O [v/v] and 1% APTES [v/v]) and incubated at RT for 30 min. Subsequently, slides were washed three times for 5 min in acetone, dried in a N_2_ stream and incubated for 45 min at 110 °C. After incubation, slides were first cooled down to RT using an N_2_ gas stream and then put into a p-phenylene diisothiocyanate (PDITC, Sigma-Aldrich, Germany) solution (10 mM PDITC in 90% DMF [v/v] and 10% pyridine [v/v]), followed by an incubation for 2 h at RT. Subsequently, the slides were flushed with ethanol and washed two times in ethanol for 5 min, followed by acetone for 5 min. Slides were dried in a N_2_ stream and put under vacuum in a desiccator for 15 min. Thereafter, slides were primer coupled by incubation in a primer solution (150 mM NaH2PO4, 150 mM Na2HPO4, 200 nM aminated DNA primer (Biomers, Germany)) at RT overnight. After incubation, slides were put into a blocking solution (10 mg/ml BSA in water) for 5 min. Then, 5% [v/v] ethanolamin was added and slides were incubated for another 25 min. Subsequently, slides were rinsed with water several times and put into 70 °C hot water for 10 min. The hot water was exchanged and slides were incubated for 5 min at 70 °C, rinsed with water at RT and dried in an N_2_ gas stream.

### Production of primer coupled PDMS master cavity chips

PDMS cavity chips were rinsed with 70% denatured ethanol, dried in an N_2_ gas stream and put under vacuum in a desiccator for 30 min. Thereafter, chips were plasma activated using a plasma generator (ZEPTO, Diener electronics) for 1 min at 30 W and at a gas flow of 20 l/h. Subsequently, chips were put into a (3-Aminopropyl)triethoxysilane (APTES) solution (90% ethanol [v/v], 5% dH2O [v/v], 5% APTES [v/v]) overnight. After incubation, chips were washed three times in ethanol for 5 min, dried in an N_2_ gas stream and incubated for 45 min at 70 °C. Next, chips were cooled down to RT using an N_2_ gas stream and put into a p-phenylene diisothiocyanate (PDITC) solution (10 mM PDITC in 90% DMF [v/v] and 10% pyridine [v/v]) followed by an incubation at RT for 2 h. Thereafter, chips were washed three times in ethanol for 5 min, dried in an N_2_ stream and put under vacuum in a desiccator for 1 h. After incubation, chips were put out of vacuum one after another and filled with a primer solution (150 mM NaH2PO4, 150 mM Na2HPO4, 2 µM aminated DNA primer (Biomers, Germany)) by adding 10 µl of solution directly onto the cavity region. Cavity chips were closed using a clean, untreated microscope glass slide and incubated at RT overnight. After incubation chips were put into a blocking solution (10 mg/ml BSA in DI water) and put under vacuum until all cavities were properly filled with blocking solution. Subsequently, chips were taken out of the vacuum and incubated for 5 min. Then, 5% [v/v] ethanolamin was added and chips were incubated for another 25 min. Next, chips were rinsed with water several times and put into 70 °C hot water for 10 min. The hot water was exchanged and chips were incubated 5 min at 70 °C, rinsed with water at RT and dried in an N_2_ gas stream.

### DNA microarray copy

#### PCR 1 - Scanning of the original DNA microarray

Primer coupled PDMS master cavity chips were filled with 4.8 µl of spPCR mix (5 U Taq-polymerase [QIAGEN, Germany], 1x Taq reaction buffer, 1.5 mM MgCl_2_, 0.6 mM dNTPs [QIAGEN, Germany], 3 mg/ml BSA, 0.05% Tween 80 [v/v], 0.125 µM forward DNA primer, 2 µM reverse DNA primer) and sealed with a glass slide containing the original DNA microarray. Next, the outer chip regions of the closed chips were filled with 50% glycerin. The microfluidic holes, as well as the backside region of the cavity region, were covered with 1 mm thick PDMS pieces. An untreated glass slide was placed onto the PDMS pieces and the whole copy chip sandwich was clamped between a small, U-shaped aluminium holder, containing four plastic spring screws. Thereafter, a standard PCR was performed using three temperature regulated water baths (Julabo, Germany) for 15 cycles. Then, the chip stacks were opened and the chips as well as the original DNA microarrays were washed in 5x SSC buffer, containing 0.1% SDS for 5 min, followed by a washing step in 0.1x SSC buffer for 5 min. Next, the glass slides were flushed with water and dried in an N_2_ gas stream. The PDMS master cavity chips were dehybridised at 95 °C for 5 min in a dehybridisation solution (50% urea [v/v], 0.5% Tween 20, 340 mM NaCl in water). Subsequently, chips were washed with water and dried in an N_2_ gas stream.

#### PCR 2 - Blocking of the PDMS master cavity chip cavities

The whole process of PCR 1 was repeated in order to block all remaining primer on the PDMS master cavity chip. However, for this step a blocking spPCR mix (containing 2 µM DNA blocking oligo instead of primer) and normal, untreated glass slides instead of an existing microarray were used. The blocking oligos consisted of the sequence “CCCCCCATGCGGGGGGTAGGTCCTA”, followed by the reverse complementary sequence of the primer on the cavity chip surface. After the PCR and disassembling of the chip stacks, the untreated glass slides used to close the cavities were thrown away instead of washed and dried.

#### PCR 3 - DNA microarray copy

The blocked and dehybridised PDMS master cavity chips were put under vacuum in a desiccator for 30 min. Then, the cavity chips were taken out of the vacuum one after another, cavities were filled with 4.8 µl of spPCR mix and sealed with primer coupled glass slides. These slides contained the same forward PCR primer sequences attached to their surfaces as those used in the spPCR mix. Again, the closed chips were filled with 50% glycerin and the microfluidic holes, as well as the backside region of the cavity region, were covered with 1 mm thick PDMS pieces. An untreated glass slide was placed onto the PDMS pieces and the whole copy chip sandwich was clamped between a small, U-shaped aluminium holder, containing four plastic spring screws. Thereafter, a standard PCR was performed using three temperature regulated water baths for 15 cycles. Then, the chip stacks were opened and the chips as well as the original DNA microarrays were washed in 5x SSC buffer, containing 0.1% SDS [v/v], for 5 min, followed by a washing step in 0.1x SSC buffer for 5 min. Next, the chips and glass slides, which then were a copy of the original DNA microarray, were rinsed with water and dried under an N_2_ gas stream.

### Glass slide hybridisation and fluorescent measurement

Glass slides were washed in 5x SSC buffer, containing 0.1% SDS [v/v], for 5 min, followed by a washing step in 0.1x SSC buffer for 5 min. Afterwards, slides were dehybridised in dehybridisation solution (50% urea [v/v], 0.5% Tween 20, 340 mM NaCl in water) for 5 min at 95 °C, followed by a washing step in water for 5 min at RT. Next, slides were hybridized (5x SSC buffer, 0.1% SDS, 10 nM Cy5 labelled DNA probe (Biomers, Germany), 10 nM Cy3 labelled DNA probe (Biomers, Germany)) for 5 min at 95 °C. Subsequently, slides were put to 40 °C together with the hybridisation solution for 10 min. Thereafter, slides were washed in 2x SSC, containing 0.1% SDS [v/v], for 3 min at 40 °C, followed by a washing step in 1x SSC for 5 min at 40 °C. Next, slides were rinsed in water and dried in an N_2_ gas stream. Afterwards, fluorescent signals of the hybridized DNA probes were analysed in a GenePix 4000B microarray scanner using GenePix software version 7 Pro. All further analyses regarding spot intensities were made using ImageJ 1.51 s.

### DNA binding analysis

All SCORE experiments were performed with our in-house build SCORE machine, which was previously published^18^. The exposure time of the camera was set to 19 ms with an image averaging of 64. We used a flow cells made of PDMS with a microfluidic gap of 30 μm and a total volume of 25 μl. Experiments were analysed using Anabel^27^.

#### Cas9 assay

The slides were blocked with 1 ml of 10 mg/ml BSA in Cas9 reaction buffer (20 mM HEPES, 0.1 M NaCL, 5 mM MgCl_2_, 0.1 mM EDTA, pH 6.5) for 10 min. After washing with DI water, the slides were dried in an N_2_ stream and inserted into the SCORE setup. The following microfluidic assay sequence was performed: (1) Cas9 reaction buffer (300 s; 60 μl/min); (2) 10 mg/ml BSA in Cas9 reaction buffer (300 s; 60 μl/min); (3) Cas9 reaction buffer (300 s; 60 μl/min); (4) 48 ng/µl apo-dCas9 in Cas9 reaction buffer (400 s; 60 μl/min); (5) Cas9 reaction buffer (900 s; 60 μl/min)

#### Thrombin assay

The slides were de-hybridised (50% urea [v/v], 0.5% Tween 20, 340 mM NaCl) for 5 min at 95 °C. Thereafter, slides where blocked with 1 ml of 10 mg/ml BSA in BBKC buffer (20 mM TRIS, 100 mM NaCl, 2 mM MgCl_2_, 5 mM KCl, 1 mM CaCl_2_, 0.02% Tween20 [v/v], pH 7.6) for 10 min. After washing with DI water, the slides were dried in an N_2_ stream and inserted into the SCORE setup. The following microfluidic assay sequence was performed: (1) BBKC buffer (300 s; 60 μl/min); (2) 10 mg/ml BSA in BBKC buffer (300 s; 600 μl/min); (3) BBKC buffer (300 s; 60 μl/min); (4) 10 µg/ml Thrombin (Sigma-Aldrich, Germany) (300 s; 60 μl/min); (5) BBKC buffer (600 s; 60 μl/min); (6) 10 µg/ml anti-thrombin antibody (ab20877, Abcam, Germany) (300 s; 60 μl/min); (7) BBKC buffer (200 s; 60 μl/min); (8) 10 µg/ml secondary antibody (ab150110, Abcam, Germany) (300 s; 60 μl/min); (9) BBKC buffer (200 s; 60 μl/min)

### Statistical analyses

Statistical analyses of DNA microarray spots were performed using ImageJ 1.51 s. Illustrated data values are expressed as means ± standard deviation if not stated otherwise. Ratio values of fluorescent intensities between the original and copied microarrays were calculated by making use of the propagation of uncertainty. The supplementary movies S4 and S5 were created by using three different ImageJ macros (Supplementary Software S6–S8).

## Supplementary information

Supplementary Information

Supplementary Movie S4

Supplementary Movie S5

## Data Availability

The tiff images and SCORE datasets of the current study are available from the corresponding author on reasonable request. Master and diploma theses are available as PDFs from the corresponding author on request.
