# Supplementary material for: How to copy and paste DNA microarrays
Source: Sci Rep. 2019 Sep 26;9:13940. doi: 10.1038/s41598-019-50371-1 (PMC6763488; doi:10.1038/s41598-019-50371-1)
Supplement: Supplementary file 1 — Supplementary Information [file 41598_2019_50371_MOESM1_ESM.docx]

Supplementary Materials

How to copy and paste DNA microarrays

Stefan D. Krämer^†,1,2*^, Johannes Wöhrle^†,1,3^, Philipp A. Meyer^1,3^, Gerald Urban^3,5^, Günter Roth^1,2,4,5^

1 ZBSA – Center for Biological Systems Analysis, University Freiburg, Freiburg, Germany

2 Faculty for Biology, Biology 3, University Freiburg, Freiburg, Germany

3 IMTEK – Dep. of Microsystems Engineering, University of Freiburg, Freiburg, Germany

4 BioCopy GmbH, Freiburg, Germany

5 BIOSS – Center for Biological Signalling Studies, University Freiburg, Freiburg, Germany

† Authors contributed equally to this work

* Corresponding Author: stefan.kraemer@zbsa.uni-freiburg.de

**Supplementary Table S1:** PDMS cavity chip dimensions of the used cavity chips.

|  | Chip 1 | Chip 2 | Chip 3 |
| --- | --- | --- | --- |
| Dimensions | 16 mm x 10 mm | 16 mm x 10 mm | 16 mm x 10 mm |
| Number of cavities | 4,104 (54×76) | 1,152 (36×32) | 476 (28×17) |
| Cavity diameter | 150 μm | 300 μm | 500 µm |
| Cavity spacing | 50 μm | 50 μm | 50 µm |
| Cavity volume | 530 pl | 2.12 nl | 5.8 nl |
| Cavity depth | 30 μm | 30 μm | 30 μm |

**Supplementary Figure S2:** **Representation of all microarray copy pictures of the “ZBSA” copy experiment.** Fluorescent intensity values of all pictures were increased by a factor of 4 in the green and by a factor of 7 in the magenta channel for better visualization. * Copies show lower signal compared to others of the same chip group probably caused by surface irregularities.


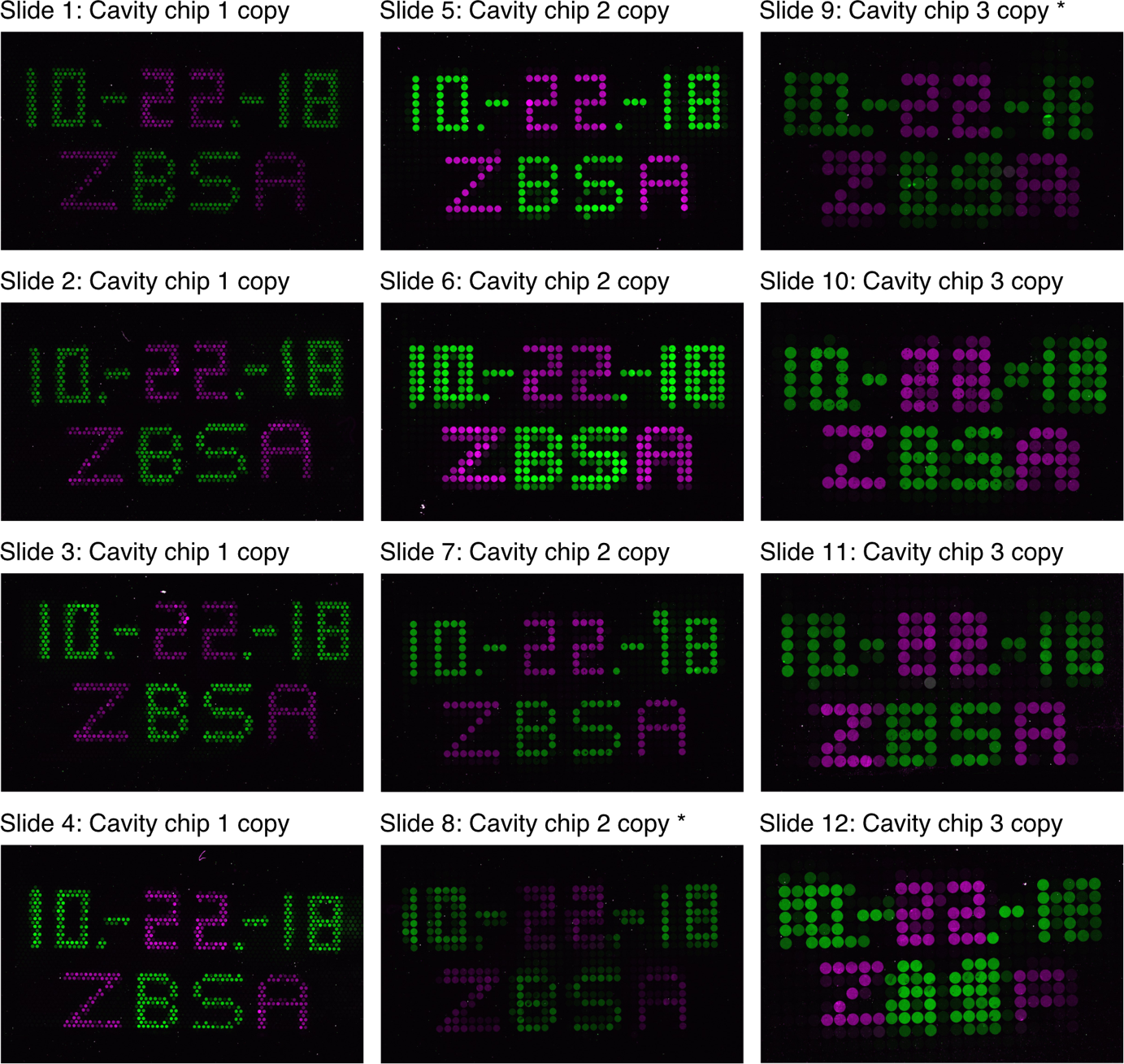


**Supplementary Figure S3: Fluorescent analysis of original and copied microarrays.** Fluorescent values were measured for each spot of the copied and original microarray using imageJ. The median signal intensities were devided by the corresponding slide background (a larger region close to the spots). We used slide 5-7 of the copied slides and their corresponding original slides for the analysis (Supplement figure 1). We excluded slide 8 from the analysis, since its signal intensities are 2.5 times smaller compared to the average signal intensities which is a sign of an artificially bad surface chemistry. Including this slide would result in artificial fluorescent values. Therefore, the number of replicates in this illustration is 3 for each group. Error bars represent the standard deviation.

**
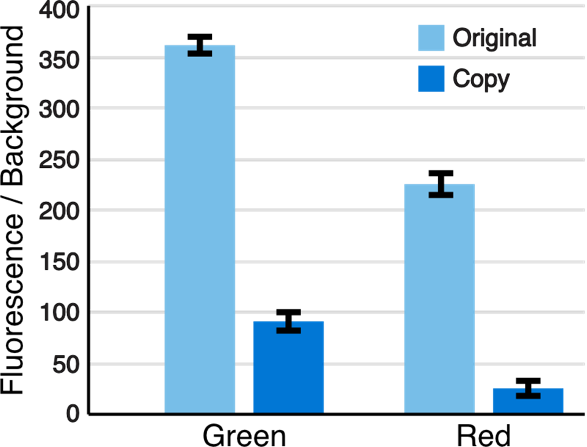
**

**Supplementary Movie S4:** **Composite movie of the apo-dCas9 SCORE binding experiment.** The left slide of the movie displays the original, whereas the right side shows its corresponding copy. Timepoints of reagent injections are indicated in the video.

**Supplementary Movie S5:** **Composite movie of the Thrombin SCORE binding experiment.** The left slide of the movie displays the original, whereas the right side shows its corresponding copy. Timepoints of reagent injections are indicated in the video.

**Supplementary Software S6: SCORE movie maker.** The following ImageJ macro was used to create an image sequence from SCORE raw data images. The SCORE raw images need to be .fts files. We used the following parameter for our SCORE videos: First Picture = 500**;** Last Picture = Last picture of SCORE raw data series (automatically)**;** Reference Picture = 620**;** Min Contrast = 0.995**;** Max Contrast = 1.050

*path = getDirectory("Choose the folder containing SCORE raw data");*

*list = getFileList(path);*

*counter = 0;*

*for (i = 0; i < list.length; i++) {*

*if (indexOf(list[i], ".fts") >= 0) {*

*counter = counter + 1;*

*}*

*};*

*width=512; height=512;*

*Dialog.create("New Image");*

*Dialog.addCheckbox("Inverted: ", true);*

*Dialog.addNumber("First Picture: ", 1);*

*Dialog.addNumber("Last Picture: ", counter);*

*Dialog.addNumber("Reference Picture: ", 0);*

*Dialog.addMessage("FTS Pictures in Folder: " + counter);*

*Dialog.addNumber("Min Contrast: ", 0.995)*

*Dialog.addNumber("Max Contrast: ", 1.05)*

*Dialog.show();*

*inverted = Dialog.getCheckbox();*

*first_pic = Dialog.getNumber();*

*last_pic = Dialog.getNumber();*

*ref_pic = Dialog.getNumber();*

*min_cont = Dialog.getNumber();*

*max_cont = Dialog.getNumber();*

*save_path = getDirectory("Select an EMPTY folder:")*

*for(i=ref_pic-3; i< ref_pic+3; i++){*

*open(path + "/" + list[i]);*

*run("32-bit");*

*run("Add...", "value=32500");*

*}*

*run("Images to Stack", "name=Stack title=[]");*

*run("Z Project...", "projection=Median");*

*rename("ref");*

*selectWindow("Stack");*

*close();*

*saveAs("Tiff", save_path+"/" + "ref.tif");*

*close();*

*open(save_path+"/"+"ref.tif");*

*for (i = first_pic-1; i < last_pic+1; i++) {*

*if (indexOf(list[i], ".fts") >= 0) {*

*for(j=i; j< i+5; j++){*

*open(path + "/" + list[j]);*

*run("32-bit");*

*run("Add...", "value=32500");*

*rename("1");*

*}*

*run("Images to Stack", "name=Stack title=1 use");*

*run("Z Project...", "projection=Median");*

*rename("1");*

*selectWindow("Stack");*

*close();*

*if(inverted == true){*

*imageCalculator("Divide create 32-bit", "1","ref.tif");*

*selectWindow("Result of 1");*

*}*

*else{*

*imageCalculator("Divide create 32-bit", "ref.tif","1");*

*selectWindow("Result of ref.tif");*

*}*

*run("Median...", "radius=2");*

*setMinAndMax(min_cont, max_cont);*

*run("Royal");*

*wait(100);*

*saveAs("Jpeg", save_path+"/" + i + ".jpg");*

*wait(100);*

*close();*

*selectWindow("1");*

*close();*

*}*

*};*

**Supplementary Software S7:** **SCORE movie picture scaling.** The following ImageJ macro was used to reduce the picture sizes of the movie images produced using supplement software 1.

*path = getDirectory("Choose the folder");*

*list = getFileList(path);*

*save_path = getDirectory("Select an EMPTY folder:")
;*

*for (i = 0; i < list.length; i++) {*

*open(path + "/" + list[i]);*

*run("Flip Vertically");*

*run("Size...", "width=720 height=607 constrain average interpolation=Bilinear");*

*wait(100);*

*saveAs("Jpeg", save_path+"/" + list[i]
);*

*wait(100);*

*close();*

*};*

**Supplementary Software S8: SCORE movie stitching.** The following ImageJ macro was used to stitch two movies together in order to generate a composite movie. Thereafter, the actual movie was created by importing the resulting images into ImageJ as “Image sequence” and using the “virtual stack” option. Subsequently, the movie was saved as an .avi file.

*score_path = getDirectory("select movie images 1");*

*binding_path = getDirectory("select movie images 2");*

*score_list = getFileList(score_path);*

*binding_list = getFileList(binding_path);*

*save_path = getDirectory("Select an EMPTY folder:");*

*for (i=0; i<score_list.length; i++) {*

*score_list[i] = parseInt( replace( score_list[i], ".jpg", "" ) );*

*}*

*Array.sort( score_list );*

*for (i=0; i<score_list.length; i++) {*

*score_list[i] = toString(score_list[i])+".jpg";*

*}*

*for (i=0; i<binding_list.length; i++) {*

*binding_list[i] = parseInt( replace( binding_list[i], ".jpg", "" ) );*

*}*

*Array.sort( binding_list );*

*for (i=0; i<binding_list.length; i++) {*

*binding_list[i] = toString(binding_list[i])+".jpg";*

*}*

*for (i = 0; i < score_list.length; i++) {*

*open(score_path + "/" + score_list[i]);*

*rename("1");*

*makeText("Original", 15, 30);*

*run("Properties... ", "stroke=white font=30");*

*run("Add Selection...");*

*run("Select None");*

*open(binding_path + "/" + binding_list[i]);*

*rename("2");*

*makeText("Copy", 15, 30);*

*run("Properties... ", "stroke=white font=30");*

*run("Add Selection...");*

*run("Select None");*

*run("Images to Stack", "method=[Copy (top-left)] name=Stack title=[]");*

*run("Make Montage...", "columns=2 rows=1 scale=1");*

*saveAs("Jpeg", save_path+"/" + score_list[i]);*

*close();*

*close();*

*}*
